# Influence of the length of the first and second metatarsal bone measured distal from Maestro line upon severity of hallux valgus deformity

**DOI:** 10.1038/s41598-021-91085-7

**Published:** 2021-06-02

**Authors:** Mirko Sovilj, Andreja Baljozović, Zoran Baščarević

**Affiliations:** 1 Hospital “Dr Mladen Stojanović”, Prijedor, Bosnia and Herzegovina; 2 Institute for Ortopedics “Banjica, Belgrade, Serbia; 3 Faculty of Medicine, Belgrade, Serbia

**Keywords:** Anatomy, Medical research, Pathogenesis

## Abstract

To examine the influence of the configuration of the first and second metatarsal (MT) bones on the development of hallux valgus deformity. To determine the extent to which the difference in the lengths of the first and second MT bones, measured distal to the Maestro line, contribute to the severity of the hallux valgus (HV) deformity defined by the size of the hallux valgus angle (HVA) and inter-metatarsal angle (IMA). On a sample of 319 feet with HV deformity the difference of measured lengths R = d (I MT) − d (II MT) was calculated The influence of differences (R) on the values of IMA and HVA as well as on the severity of deformities according to the formed groups was investigated. The influence of age on the development of deformities was examined separately as well as in conjunction with the determined difference in lengths. In 203 feet or 63.7%, a shorter MT bone was measured, while in 80 feet or 25.1% the first MT bone was longer than second ones and only in 36 feet or 11.3% there is no difference in the length of the 1st and 2ndMT bones distal to the Maestro line. A statistically significant correlation was found between the difference between the measured lengths of 1st and 2nd MT bones and IMA, while this correlation with HVA was not statistically significant. There is no statistically significant correlation or the effect of the difference in measured lengths (R) on the severity of hallux valgus deformity classified into three groups. A statistically significant correlation and impact of the age on the intensity of the deformity are established. A shorter 1st MT bone in correlation to the 2nd MT bone is accompanied by an increase in IMA and this correlation and impact are statistically significant. It was not established that there was a statistically significant influence of the length of the first and second metatarsal bone measured distal from Maestro line upon the values of HVA and severity of HV deformity. Age significantly contributes to the severity of the deformity.

## Introduction

Genetic predisposition is the most common explanation for the occurrence of hallux valgus deformity and according to research; about 70% to 90% of patients confirm the presence of this deformity in the family^1–3^. However, a special place in the study of endogenous etiological factors is occupied by the configuration of the front part of the foot, which is defined by digital and metatarsal formula^3,5^. The shape of the head of the first metatarsal bone and its articular surface, as well as the torsion of this bone, contribute to the assessment of the influence of endogenous factors on the occurrence and progression of hallux valgus deformity^6–8^. A very small number of papers are devoted to the influence of bone and joint structure, which as such is genetically determined, on the development of hallux valgus deformity. They show that the measured lengths of the first (I MT) and second metatarsal (II MT) bones were analyzed as relative values. The correlation between the levels of articular surfaces of 1st and 2ndMT bones was determined using the Morton transverse line or using the Hardy–Clapham arc line^1,5,9,10^. The results of published papers indicate that the excessive length of the first metatarsal bone compared to the second is more often associated with hallux valgus^3,11^, than when it is shorter^12^. The research published so far is based on different methods of measuring the level of the head of the first and second metatarsal bones and the established correlation was only related to the occurrence of hallux valgus deformity, without investigating the impact on its severity^1,5^.

In order to determine the ratio of the articular surfaces of the first and second metatarsal bone, in this work we measured the length of 1st and 2nd metatarsal bone distal to the Maestro line. This line is the basis for determining the configuration of the anterior segment of the foot and the possible deviation from the metatarsal parabola according to Maestro's criteria^4,13,14^. Thus, the main goal of this study is to determine whether and to what extent the difference in the length of the first and second metatarsal bones distal to the Maestro line affects the severity of hallux valgus deformity or the size of the hallux valgus angle and inter-metatarsal angle and whether this influence in correlation to the age of the respondents.

## Material and methods

The conducted observational research is a combined descriptive-analytical study which treated a total of 269 patients, 396 operated feet with severe hallux valgus deformity who were treated at the Institute for Orthopedic Surgical Diseases "Banjica" in Belgrade in the period from 1993 to 2010. At the time of admission, all patients agreed that the medical records of their treatment could be used for research purposes. The informed consent for subjects under 18 years was obtained from their parents. All applied aspects of the study were approved by the institution. The consent of the Ethics Committee of the Institute for this study was also obtained.

**Figure 1:**
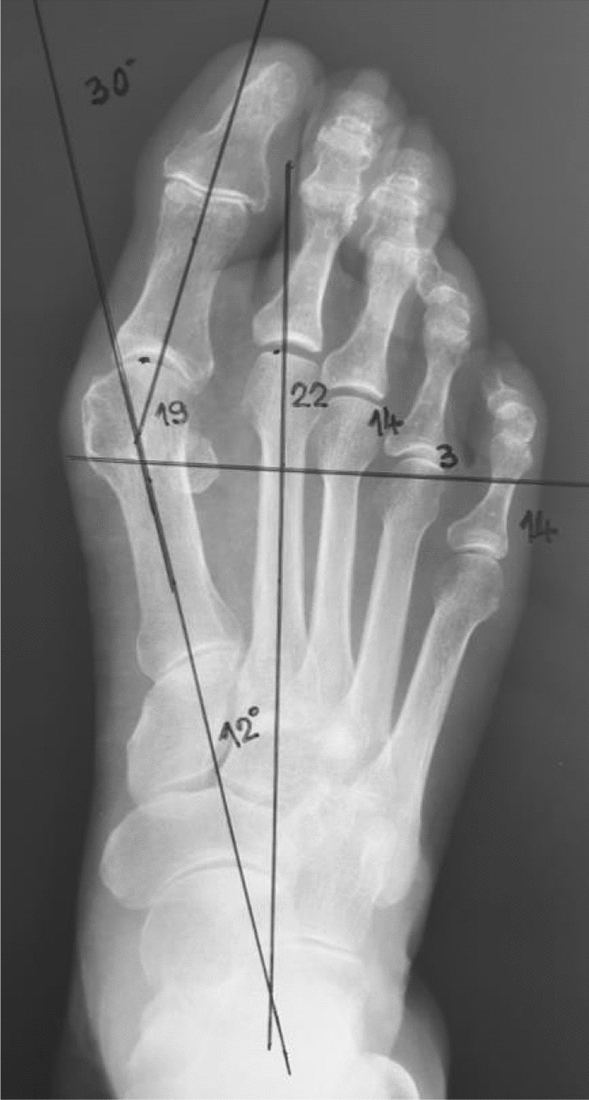
The measured length of the 1st and 2nd metatarsal bones distal from Maestro line.

For the needs of preoperative planning, radiography of the feet was performed with a load with an inclined X-ray tube of 15 degrees in correlation to the vertical and at a distance of 1 m. HVA and IMA measurements were performed on the X-ray film, as well as additional measurements of metatarsal bone length in correlation to the Maestro line. (Fig. 1).

Cases that have previously undergone osteoarticular surgical treatment or have previously had injuries to the bone and joint structures of the foot, suffering from rheumatism, diabetes, or having neuromuscular disease are excluded from this study. Based on the stated criteria, 77 cases were excluded, so that further analysis was performed on 319 feet.

In this study, measurements of the 1st and 2nd metatarsal bones were performed distal to the Maestro line, which is drawn perpendicular to the 2nd MT bone axis at the level of the middle of the lateral sesamoid and which usually passes through the central part of the head of the fourth metatarsal bone (SM4 axis). By measuring the articular surface of the heads in correlation to the Maestro line, a metatarsal parabola is obtained which, as a normal finding, defines equal length of 1st and 2nd MT bones and then successive shortening of MT bones by geometric progression with coefficient 2 (3 mm, 6 mm, 12 mm)^4,15^. Accidental errors in this study were minimized and were caused by radiographic imaging according to the established methodology and possibly by unintentional measurements made by the author^5^.

Out of 319 treated feet that were surgically treated for severe hallux valgus deformity in the observed period, 316 or 99.1% belonged to women and only 3 feet or 0.9% to men. The mean age of the patients enrolled in the study was 45.75 years with a standard deviation of 13.08 years. For the purposes of the analysis, five categories were formed in correlation to the age of the patients at the time of the operation, namely:

$$\begin{array}{ll} {Minors\;\left( { \le 18\;{\text{years}}} \right)} & {9\;{\text{cases}}\;{\text{or}}\;2.8\% ,} \\ {Younger\;age \, \left( {19 - 32} \right)} & {41\;{\text{cases}}\;{\text{or}}\;12.9\% ,} \\ {Middle\;age \, \left( {33 - 46} \right)} & {103\;{\text{cases}}\;{\text{or}}\;32.3 \, \% ,} \\ {Older\;age \, \left( {47 - 59} \right)} & {119\;{\text{cases}}\;{\text{or}}\;37.3\% ,} \\ { \ge 60\;years} & {47\;{\text{cases}}\;{\text{or}}\;14.7\% ,} \\ \end{array}$$

The severity of hallux valgus deformity was determined by the values of hallux valgus angle (HVA> 15) and inter-metatarsal angle between the first and second MT bone (IMA> 9) and accordingly we formed three groups^16^, as follows:

- g1*: mild deformity* (HVA<30 & IMA<13);
- g2: *moderate deformity* (HVA30 - 40 & IMA 13-20);
- g3: *severe deformity* (HVA>40 & IMA >20);

Obtained values of measured differences in the lengths of the first and second metatarsal bones distal to the Maestro line (R = d (1st MT) − d (2nd MT)) are classified into three broader groups:

- *R* > 0, the first metatarsal bone is longer than the second one (index plus tip metatarsal formula).
- *R* = 0, the first metatarsal bone is equal to the second one (index plus-minus tip metatarsal formula).
- *R* < 0, the first metatarsal bone is shorter than the second one (index minus tip metatarsal formula) (Fig. 1).

Descriptive statistical analysis calculated the corresponding statistical indicators of continuous and categorical variables. The Pearson and Spearman correlations were used to estimate the strength of the correlation between the two variables. The difference between the groups of hallux valgus deformities depending on the ratio of 1st and 2nd MT bone lengths distal to the Maestro line and the age of the patients was evaluated by the Kruskal-Wallis test, while the difference between the groups was evaluated by the Mann-Whitney U test. To assess the influence of one or two factors on the deformity, a one-factor or two-factor analysis of variance was used. Tukey HSD test was also used to identify the significance of the actual differences between the groups. The importance of the obtained results was assessed by the strength of the connection by calculating the indicators of the partial eta of the square. Statistical analysis was done using a software package IBM SPSS Statistics 23.

### Ethical approval

The authors confirm that informed consent was obtained from all subjects. The informed consent for subjects under 18 years was obtained from their parents. The authors confirm that all research protocols were approved by the licensing comitte of the Institute for Ortopedics “Banjica Belgrade”, Serbia. The authors confirm all methods were carried out in accordance with relevant guidelines and regulations. The images included in the manuscript were not created using any software.

## Results

First, the basic descriptive indicators of the dispersion of the results of the measured quantities whose correlation s we want to analyze are determined (Table 1). The frequency of the calculated difference between the first and second metatarsal bones distal to the Maestro line was then analyzed (R).

**Table 1:** Values of descriptive variables.

Variables	N	Min.	Max	Mean (std.error)	S. D	Skewness (std. error)	Kurtosis (std. error)
AGE	319	13	73	45.75(0.731)	13.05	− 0.307 (0.137)	− 0.524 (0.272)
HVA	319	16	57	33.43 (0.409)	7.313	0.169 (0.137)	0.165 (0.272)
IMA	319	10	28	13.84(0.168)	2.995	0.999 (0.137)	1.992 (0.272)
R	319	– 12	12	− 1.4169 (0.182)	3.250	0.236(0.137)	0.792(0.272)

Hallux valgus angle (HVA), Inter-metatarsal angle (IMA), Difference (*R* = *d*(1st *MT*) − *d*(2nd *MT*)).

**Figure 2:**
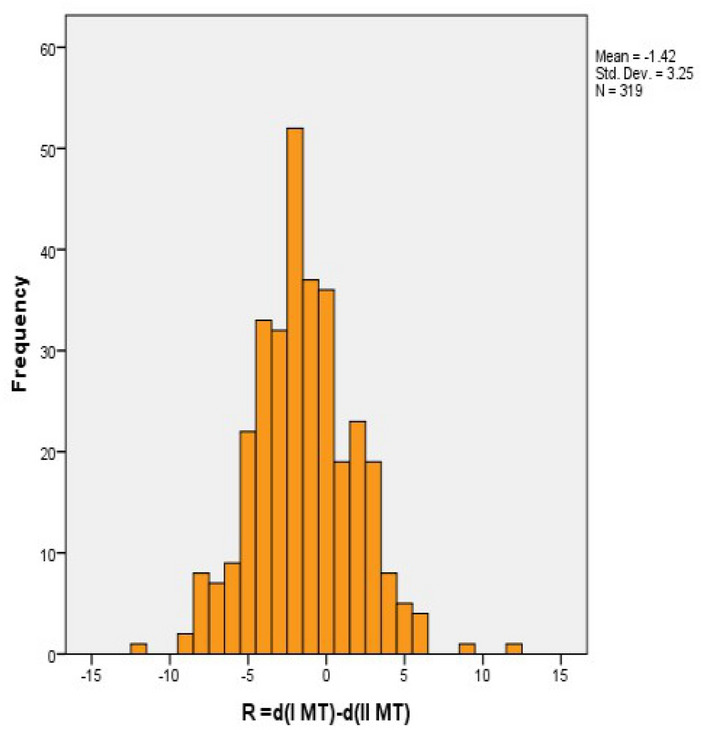
Graphical representation of the length difference distribution.

Of the 319 feet analyzed, in 80 (or 25.1%) the first MT bone was longer than the second one, measured distal to the Maestro line (R > 0), while the same measurement in 36 feet (or 11.3%) was recorded that the lengths of the first and second MT bones are equal (R = 0). In most cases, the first MT bone is shorter than the second one, 203 or 63.7% (R < 0). On the histogram of the differences shows that the length distribution does not deviate much from the normal distribution (Fig. 2).

Calculated differences between measured lengths (R) for the purposes of analyzing correlations have been classified into five narrower groups:

- R1: (R, ≤ − 6 mm): the first MT bone is *significantly shorter* from the second one, 27 cases (or 8.5%),
- R2: (− 6 mm < R < 0 mm) the first MT bone is *insignificantly shorter* from the second one, 176 cases (or 55.2%),
- R3: (R = 0): the first MT bone is *equal* to the second one, 36 cases (or 11.3%),
- R4: (0 mm < R < 6 mm): the first MT bone is *insignificantly longer* from the second one, 74 cases (or 23.2%), and
- R5: (R ≥ 6 mm): the first MT bone is *significantly longer* from the second one, 6 cases (or 1.9%).

**Figure 3:**
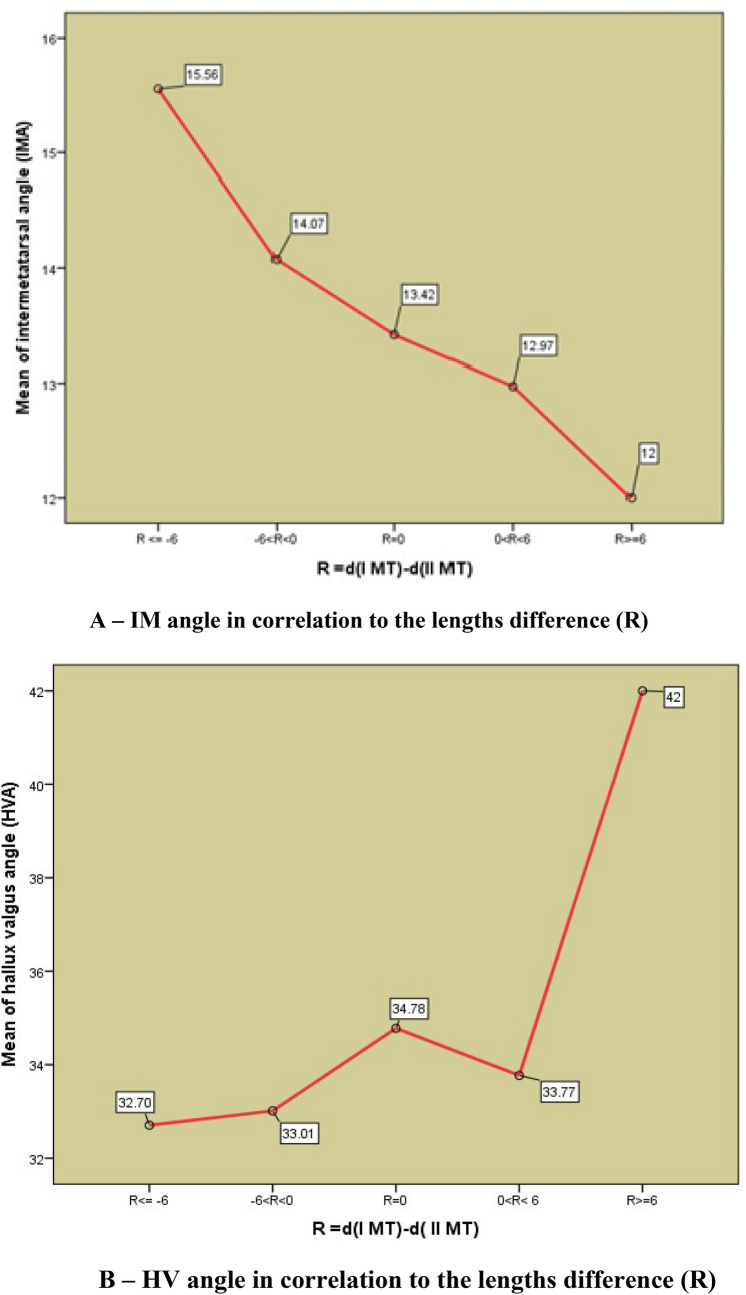
Graph of median values: (**A**) IMA in correlation to the lengths difference and (**B**) HVA in correlation to the lengths difference I and II MT bone distal to Maestro line.

Spearman rang coefficient demonstrates statistically significant correlation between the length difference (R), and values of IMA: Ro = − 0.196, Sig. = 0.001, *at the significance level p* = *0.01*, with negative sign. However, the correlation between (R) and HVA is not statistically significant: Ro = 0.096, Sig. = 0.086 (Table 2, Fig. 3)

**Table 2:** Descriptive results of the Kruskal–Wallis test applied for assessment of differences between the values of HVA and IMA in correlation with the difference R.

Difference (R)	N	Hallux valgus angle (HVA)			Intermetatarsal angle (IMA)		
		Mean	SD	Median	Mean	SD	Median
R1 significantly shorter (R < = − 6)	27	32.70	7.037	34.00	15.56	3.866	15.00
R2 insignificantly shorter (− 6 < R < 0)	176	33.01	7.179	32.00	14.07	2.845	14.00
R3 equal length (R = 0)	36	34.78	8.096	34.50	13.42	2.511	13.00
R4 insignificantly longer (0 < R < 6)	74	34.07	7.204	34.50	12.97	2.965	12.00
R5 significantly longer (R > = 6)	6	32.83	9.766	35.00	12.67	2.251	12.50
Total	319	33.43	7.313	33.00	13.84	2.995	13.00
K-V test statistics		χ^2^(4, n = 319) = 2.546, p = 0.636			χ^2^(4, n = 319) = 2.546, p = 0.002

Kruskal-Wallis test showed that there was no statistically significant difference between the values of the median HVA in the formed groups of length differences (R):(χ^2^(4,n = 319)) = 2.546; p = 0.636 > 0.05), but that there was a statistically significant difference between the values of the median IMA in the mentioned groups of length differences (R): χ^2^(4,n = 319)) = 2.546, p = 0..002 < 0.05 (Table 2).

Mann–Whitney U-test revealed a statistically significant difference between the effects of the difference in length (R) on the size of the IMA between the following groups;

- ✓ Group R1 median (Md = 15.00, n = 27) was larger than the group R3 one (Md = 13.00, n = 36), U = 333.000, z = − 2.141, Sig = 0.032 and the group R4 one (Md = 12.00, n = 74), U = 589.500, z = − 3.169, Sig = 0.002.
- ✓ Group R2 median (Md = 14.00, n = 176) was larger than the group R4 one (Md = 12.00, n = 74); U = 4883.5, z = − 3.147, Sig = 0.002.

The correlation between the severity of HV deformity classified into three groups according to the values of HVA and IMA and the representation of differences in the length of the 1st and 2nd MT bones was examined. The chi- square test showed that this correlation was not statistically significant (Sig = 0.446) (Table 3).

**Table 3:** Descriptive indicators of the frequency of differences between lengths (R) in correlation with the severity of HV deformities.

		Difference					p
		Significantly shorter (R < = −), N = 27 (8.5%)	Insignificantly shorter (− 6 < R < 0), N = 176, (55.2%)	Equal (R = 0) N = 36, (11.3%)	Insignificantly longer (0 < R < 6) N = 74, (23.2%)	Significantly Longer (R > = 6) N = 6, (1.9%)	
Deformity according to Butkovic	Mild deformity (HVA < 30: IMA < 13), N = 56 (17.6%), Me = − 1.02, SD = 3.084 95% CI − 1.84 to − 0.19, Min. = − 8, Max. = 6	4(14.8%) 7.1%	28(15.9% 50.0%	8(22.2%) 14.3%	14 (18.9%) 25.0%	2(33.3%) 3.6%	χ^2^ = (8319) = 07.873 Sig. = 0.446*a
	Moderate deformity (HVA 30 to 40; IMA 13 to 20), N = 212, (66.5%), Me = − 1.64 SD = 3.099, 95%CI:− 2.06 to − 1.22, Min. = − 9, Max. = 6	19 (70.4%) 9.0%	124 (70.5%) 58.5%	19 (52.8%) 9.0%	48 (64.9%) 22.6%	2 (33.3%) 0.9%	
	Severe deformity (HVA > 40: IMA > 20, N = 51,(16.0%), Me = − 0.9, SD = 3.926, 95%CI:− 2.01 to 0.20 Min. = − 12, Max. = 12	4 (14.8%) 7.8%	24 (13.6%) 47.1%	9 (25.0%) 17.6%	12 (16.2%) 23.5%	2 (33.3%) 0.9%	
	ANOVA	F( 2,316) = 1.555,Sig. = 0.213					

*^a^5 cells (33.3%) have expected count less than 5. The minimum expected count is 0.96.

**Figure 4:**
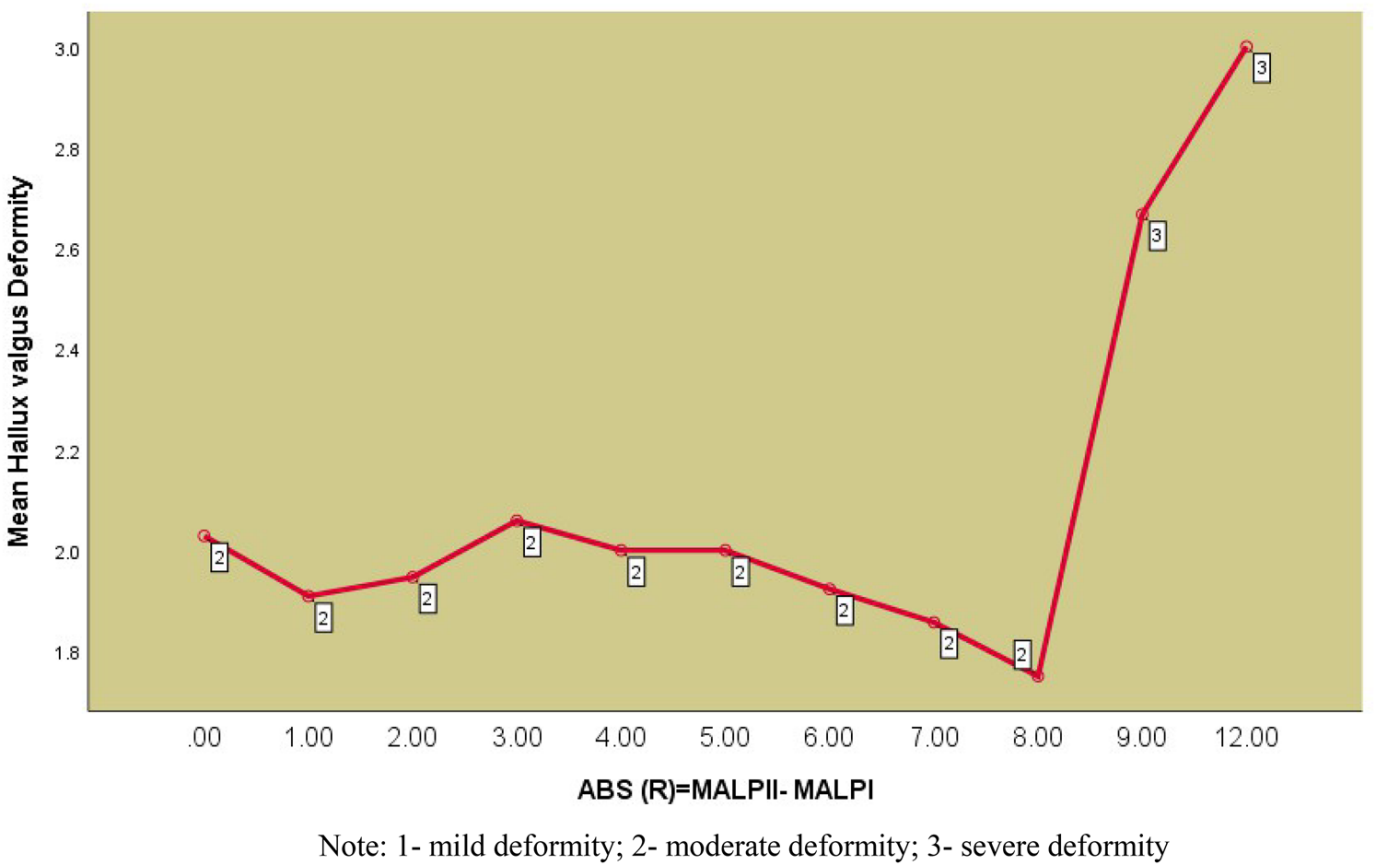
Type of deformity in correlation to the absolute value of the length difference (R). Note: 1- mild deformity; 2- moderate deformity; 3- severe deformity.

One-factor variance analysis showed that there was no statistically significant effect of the difference in 1st and 2nd MT bone lengths distal to the Maestro line on the severity of hallux valgus deformity classified into three groups (g1–g3), Sig. = 0.213, (Table 3.). Analysis of the type of deformity shows that up to 8 mm differences in the length of the first MT bone in correlation on the second one, whether it is shorter or longer, moderate HV deformity predominates (HVA 30–40, IMA13–20) and with a difference greater than 8 mm, severe deformity predominates (HVA> 40, IMA> 20) (Fig. 4).

**Figure 5:**
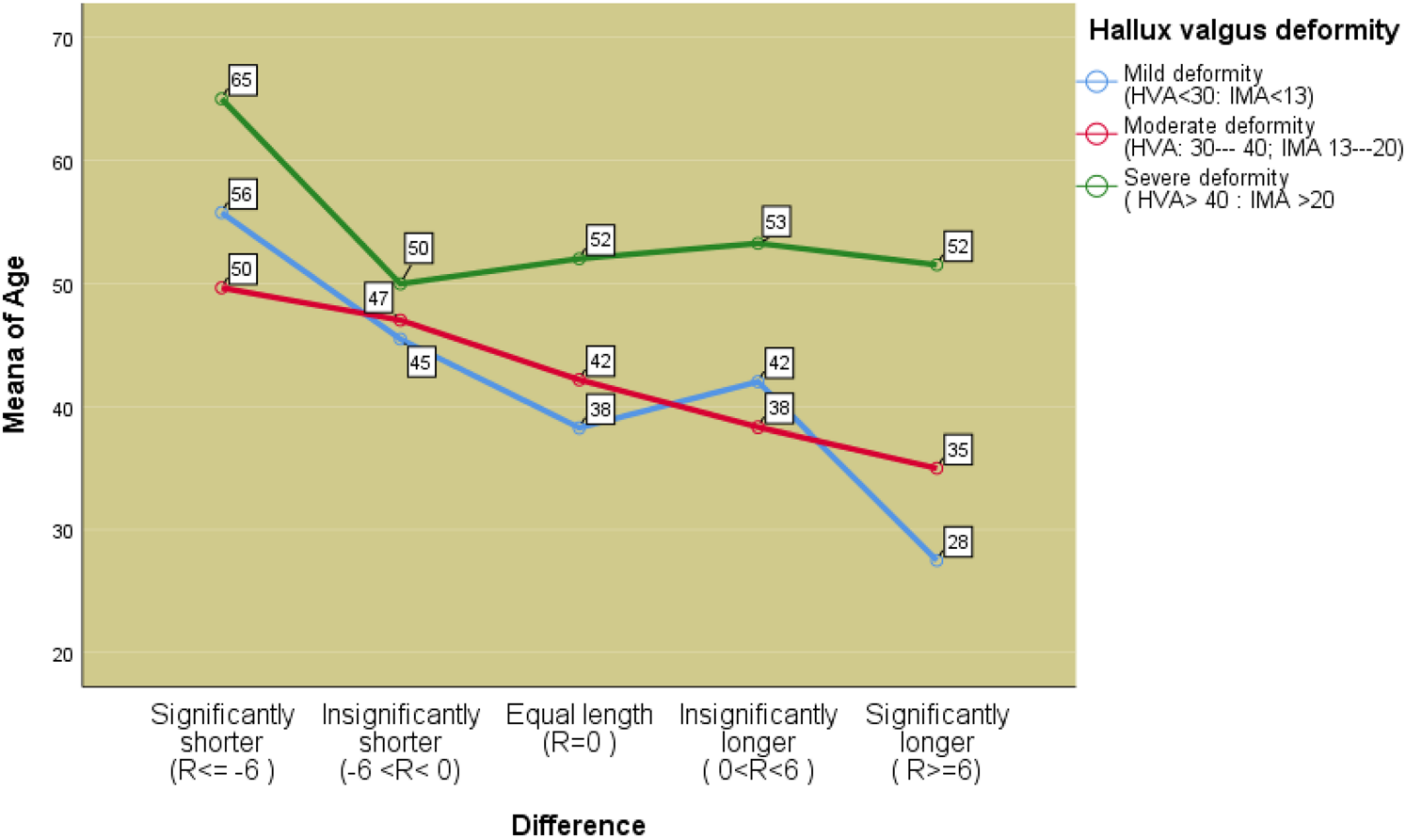
Graphic representation of the severity of deformity in correlation with the mean value of age categories and the difference in lengths of the 1st and 2nd MT bone distal to Maestro line.

For further analysis of the correlation between the length of 1st and 2nd MT bones distal to the Maestro line with the severity of hallux valgus deformity, we included the age of the patient at surgery (Fig. 5).

The chi-square independence test did not show a significant correlation between the difference in length (R) and the age category of the patient but showed that there is a statistically significant correlation between hallux valgus deformity and the age of the operated patients, χ^2^(8.319) = 25.676 Sig = 0.001.

A two-factor analysis of the variance of different groups investigated the influence of the difference between the lengths of R and the age of the subjects on the severity of the deformity. The effect of the interaction between the difference between the lengths of the first and second MT bones (R) and age was not statistically significant, F(13,297) = 1.539, Sig. = 0.102.

A statistically significant influence of age on the severity of deformity was confirmed, F (4,297) = 5.765, Sig. = 0.000, the strength of this influence can be assessed as moderate, Eta square = 0.072, while the influence of the difference between the first and the second MT bone (R) on the severity deformity (g1–g3) was not statistically significant F(4,297) = 0.132, Sig. = 0.971.

## Discussion

Out of the 319 analyzed feet that underwent surgical treatment for hallux valgus deformity, 206 (63.7%) had shorter 1st MT bone compared to the second (index minus type), measured distal to the Maestro line, of which 27 length difference 6 mm and more. In 80 feet (25.1%) I MT bone is longer than II MT (index plus type) while only in 36 feet (11.3%) there is no difference in length of 1st and 2nd MT bone (index plus / minus type, Fig. 2). These results are not in accordance with the previously published and obtained by longer measurements, using Hardy-Clapham arc line where a larger number measured with equal relative length of 1st and 2nd MT bones with 1 mm tolerance, while the same numbers of those with shorter or longer I MT bone^1^. There are also authors whose study determined the propulsion (bulge) of the head and MT bone in correlation to the second one on average 3.49 ± 3.36 mm in feet with hallux valgus deformity in correlation to the control group, without pronounced deformity, with propulsion I MT bone of 0.84 ± 3.02 mm^5^.

The value of the Spearman rank correlation coefficient showed that there was a statistically significant correlation between the length differences (R) and the IMA values (Sig = 0.001 < 0.01) and that this correlation was not statistically significant in HVA (Sig = 0.086). Kruskal–Wallis test shows that the values of the median IMA between the formed groups of differences in lengths 1st and 2ndof MT bone (R) differ statistically significantly (p = 0.002 < 0.05) but that there is no statistically significant difference in the median HVA value (p = 0.636 > 0.05) (Table 2).

The Man Whitney U-test showed that the IMA was significantly higher in the group with significantly shorter 1st MT bone (≥ − 6 mm) compared to the group in which 1st and 2nd MT bones were of equal length (Sig = 0.032) as well as in relation to the value of IMA in the group in which the 1st MT bone is slightly longer than the second one (up to 6 mm) (Sig = 0.002) and this difference is therefore statistically significant (p < 0.05). The same test revealed a significant difference in the size of the IMA in the group with slightly shorter 1st MT bone compared to the group in which the 1st MT bone was slightly longer compared to the 2nd MT bone (Sig = 0.002 < 0.05). These results cannot be compared with previously published ones because there is no data on a similar study of the influence of the ratio of lengths of the 1st and 2nd MT bone distal to the Maestro line on IMA and HVA, i.e. on the degree of hallux valgus deformity.

Thus, this analysis shows that the difference in the length of the 1st and 2nd MT bones, measured distally from the Maestro line, differently affects the basic radiographic components of the deformity—HVA and IMA. Shorter 1st MT bone is accompanied by a higher IMA between 1st and 2nd MT bone, and statistical significance was confirmed for this ratio (p < 0.01). The influence of this difference in MT lengths upon HVA did not show any statistical significance, although the increase of I MT bone length is followed by the increase of HVA. (Fig. 3).

Since the different influence of the difference between the lengths of 1st and 2nd MT bones on the basic components of HV deformity (IMA and HVA) was determined, the correlation between the severity of HV deformity classified into three groups (mild, moderate, and severe) and the presence of differences in length of the1st and 2nd MT bones classified into five groups (R1–R5) was tested. The chi-square test showed that this correlation was not statistically significant. One-factor variance analysis showed that the influence of this length difference on the severity of HV deformity classified into three groups was not statistically significant (Table 3). These results cannot be compared because such or similar research applying this method of measurement and research on the impact on the severity of deformity (i.e. combined on HVA and IMA) has not been published yet.

However, when we analyze the prevalence of type HV deformity classified according to its severity in relation to the difference in lengths of the 1st and 2nd MT bones, we notice that moderate deformity prevails with the difference ≤ 8 mm, while severe deformity prevails when it exceeds 8 mm (HVA > 40, IMA > 20).

Analysis of the influence of the difference between the lengths of 1st and 2ndMT bones on the severity of HV deformity has its limitation contained in the fact that a significant number of cases (135) were classified in the formed three groups (mild, moderate, and severe) by meeting only one criterion (value of HVA or IMA). Justification and need to apply a more precise classification of five groups for studying the influence of bone and joint structure on the development of HV deformity remains to be considered, which would enable all cases to meet both criteria of the group to which they belong. Despite the above, we believe that this study contributes to the understanding of the importance of morphology of bone and joint structures of the feet for the development of HV deformity.

Our study showed that the age has a significant contribution to development of HV deformity. The Chi-square test showed that there is a statistically significant relationship between age and the degree of deformity, and this is certainly the expected result. In addition, it was found that the influence of age on the severity of deformity was statistically significant (Sig = 0.000) and the strength of this influence was assessed as moderate (Eta square = 0.072). The effects of the interaction between the difference between the lengths of 1st and 2nd MT bones (R) and age was not shown to be statistically significant (Sig. = 0.102).

## Conclusion

Out of 319 analyzed feet with HV deformity, 63.7% of them had shorter I MT than the II MT bone measured distally from Maestro line.

A significant correlation between the difference of the I and II MT bones lengths distally from Maestro line and IMA. The shorter I MT bone is, the higher IMA.

Our study showed that the difference of the I and II MT bones lengths distally from Maestro line has not any statistically significant influence upon HVA.

We did not established statistically significant influence of the said difference of the I and II MT bones upon the severity of HV deformity.

Significant correlation between the age and HV deformity was established, as well as its influence upon severity of the deformity; while the influence of interaction between age and I and II MT bones lengths difference was not statistically significant.
